# Substance P Receptor in the Rat Heart and Regulation of Its Expression in Long-Term Diabetes

**DOI:** 10.3389/fphys.2018.00918

**Published:** 2018-07-13

**Authors:** Magdalena Chottova Dvorakova, Eliska Mistrova, Renate Paddenberg, Wolfgang Kummer, Jana Slavikova

**Affiliations:** ^1^Biomedical Centre, Faculty of Medicine in Pilsen, Charles University, Pilsen, Czechia; ^2^Department of Physiology, Faculty of Medicine in Pilsen, Charles University, Pilsen, Czechia; ^3^Institute for Anatomy and Cell Biology, Justus-Liebig-University Giessen, Giessen, Germany

**Keywords:** neurokinin receptor 1, heart, expression, distribution, diabetes mellitus

## Abstract

Substance P (SP) is a neuropeptide engaged in the signal transmission of neural C fibers afferents in the myocardium. The actions of SP in the heart are extensive and they are mediated by the neurokinin 1 receptor (NK1R), a member of the tachykinin subfamily of G-protein coupled receptors. The receptors have been found in the heart, but to our knowledge, their exact localization in the heart has not been described yet. Here, we investigated the presence of NK1R protein in separate rat heart compartments by means of western blot and its tissue distribution by means of immunofluorescence. Specificity of NK1R immunolabeling was controlled by preabsorption of the antiserum with its corresponding peptide. Additionally, we investigated abundance of gene for NK1R in separated heart chambers by means of quantitative real-time PCR (RT-PCR). Relative abundance of NK1R mRNA was expressed as a ratio of target gene Cq value to Cq value of control gene – beta-actin. Finally, we studied abundance of NK1R mRNA in different cell types of heart isolated by laser capture microdissection. Immunofluorescence showed NK1R immunoreactivity on the surface of some intracardiac neurons and smooth muscle cells of coronary vessels. The results of quantitative RT-PCR indicate abundance of mRNA for NK1R in all heart chambers with highest level in the left atrium. The presence of NK1R mRNA was detected in some samples of dissected intracardiac neurons, but not in cardiomyocytes or smooth muscle cells of coronary vessels. In the course of long-term diabetes, a significant downregulation of the NK1R mRNA was seen in the right atrium and upregulation in the right ventricle 53 weeks after the induction of diabetes. Our results indicate localization of NK1R in some intracardiac neurons and smooth muscle cells. Impaired transcription of the NK1R gene in the diabetic heart may be induced by unidentified genes or factors involved in the development of diabetic cardiomyopathy.

## Introduction

Cardiovascular autonomic neuropathy is a serious and one of the most common complications of diabetes mellitus which accompanies later stages of diabetes (Vinik et al., 2003). Its pathophysiological mechanism is attributed to damage of sympathetic and parasympathetic innervation of the heart leading, among others, to dysregulation of the neurohumoral activation of cardiomyocytes and vessels which may cause abnormalities in heart functions and vascular dynamics (Ejaz et al., 2011b). Cardiac autonomic neurons contain a broad spectrum of neurotransmitters from which long-acting neuropeptides may serve as neuromodulators or trophic factors (Burnstock, 2013). Therefore, dysregulation of their expression or stimulation of their signaling pathways can negatively impact cardiac homeostasis (Ejaz et al., 2011b). Our previous studies revealed significant changes in neurotransmission of vasoactive intestinal polypeptide and calcitonin gene-related peptide in the rat heart of experimentally induced diabetes (Chottova Dvorakova et al., 2005; Dvorakova et al., 2006). This study is focused on a further component of a neuropeptide transmission in the heart, the specific receptor for substance P (SP).

Substance P, an 11 amino acid peptide, belongs to the tachykinin family of neuropeptides which also comprises neurokinin A (NKA) and neurokinin B (NKB) (Page, 2004). In the heart, SP is localized in sensory nerve fibers derived from a special class of nociceptive neurons with cell bodies located in the nodose ganglia (Tay and Wong, 1997) and dorsal root ganglia (Papka and Urban, 1987; Corbett et al., 2005). The main targets for these unmyelinated C fibers in most mammals are the intrinsic cardiac ganglia and coronary arteries. The fibers also project to other regions of the heart including the endocardium, atrioventricular valves, nodal tissue, conducting system, and atrial contractile cells. There is also limited number of fibers in the ventricles themselves. The distribution of SP-containing nerves, however, differs between species (Schoborg et al., 2000; Dehlin and Levick, 2014). In the rat heart, SP was identified also in the cell bodies of cardiac intrinsic ganglion neurons (Steele et al., 1994; Horackova et al., 2000). Additionally, the production of this peptide was demonstrated in some non-neuronal cells such as macrophages, lymphocytes, and endothelial cells (Milner et al., 1989; Cai et al., 1993; Ho et al., 1997; Lai et al., 1998, 1999; Chang et al., 2013).

The actions of SP on the heart were studied in details in the guinea pig. They are diverse and include direct coronary vasodilatation by the release of nitric oxide, and the negative inotropic and chronotropic effects, which are mediated indirectly by the stimulation of cholinergic neurons (Chiao and Caldwell, 1995; Hoover et al., 1998, 2000). Evidence indicated that the cardiac effects of SP are primarily exerted through the neurokinin 1 receptor (NK1R), a subtype of the tachykinin G protein-coupled receptor possessing the greatest affinity for SP (Nakanishi, 1991; Maggi et al., 1993; Schoborg et al., 2000). However, information concerning to the cellular distribution of NK1R within the heart is limited and has not provided a uniform picture. Hoover and Hancock (1988) using the guinea pig as a model detected specific binding of SP in association with cardiac parasympathetic ganglia within the epicardial connective tissue adjacent to the atria, as well as coronary arteries. Specific binding of SP fails to be detected in coronary vessels or within atrial or ventricular myocardium (Hoover and Hancock, 1988). In the endothelial cells, NK1R was present in the dog carotid arteries (Stephenson et al., 1986), but absent in bovine coronary arteries (Stephenson and Summers, 1987). Walsh et al. (1996) revealed the presence of specific SP binding sites among the connective tissue skeleton of the heart, in the adventitia of the great vessels and coronary arteries, and along the cusps of both the semilunar and the atrioventricular valves in the rats. However, such binding sites were not demonstrated on the endothelial cells, cardiac muscle fibers, and smooth muscle cells. Contrary within the lung, SP was demonstrated to cause endothelium-dependent relaxation via stimulation of NK1R (D’Orleans-Juste et al., 1986; Floch et al., 1994) suggesting the presence of NK1R on endothelial cells, and SP-binding sites were found in guinea pig but not rat bronchi (Floch et al., 1994). In human, bronchi as well as pulmonary artery and vein possess NK1R mRNA (Pinto et al., 2004).

In summary, differences in the projections of SP containing nerves have been described among species and a very little is known about the distribution of SP binding sites within the heart, especially in the rat. However, accumulating evidence indicates that SP is cardioprotective due to its potent coronary vasodilator effects (Chiao and Caldwell, 1995; Ustinova et al., 1995; Wang et al., 2011). Moreover, a recent study suggests that cardioprotective role of SP performed throughout its direct actions on cardiomyocytes (Jubair et al., 2015). Therefore, the purpose of this study was to investigate the gene and protein expression of NK1R in all heart compartments of rats. In addition, the cellular distribution of the receptors was examined in the atrial and ventricular myocardium. Given the neuropeptide dysregulation in diabetes, abundance of the NK1R mRNA was studied in the model of streptozotocin (STZ)-induced diabetes lasting for 26 and 53 weeks, respectively.

## Materials and Methods

### Experimental Animals

Adult female Wistar rats purchased from VELAZ (Prague, Czechia) were used. The animals were housed six per cage. They were fed with standard laboratory chow *ad libitum* and they have free access to drinking water. Before the initiation of the experiments, the animals were left to adapt for 2 weeks. All experiments were approved by the University Committee for Experiments on Laboratory Animals and were conducted in accordance with the relevant Guidelines of the Czech Ministry of Agriculture for scientific experimentation on animals and the “Guide for the Care and Use of Laboratory Animals” (NIH Publication No. 85-23, revised 1996).

Diabetes was induced in young adults by a single injection of STZ as described previously (Chottova Dvorakova et al., 2008). Two groups of diabetic rats were studied, 26 and 53 weeks after the induction of the disease. Control animals received a corresponding volume of the vehicle. They were sacrificed 26 and 53 weeks after the injection.

Rats were anesthetized with ether and killed by decapitation. Heart was rapidly excised and directly frozen in liquid N_2_ (for Western blot and RNA isolation), or embedded in optimum cutting temperature compound (Takara, United States) and frozen in precooled isopentane (for laser microdissection and immunofluorescence). Hearts used for RNA isolation and Western blot were rinsed with ice-cold saline, freed of connective tissue and fat, and divided into the left atrium (LA) with the interatrial septum, right atrium (RA), and free walls of left ventricle (LV) and right ventricle (RV) before freezing as described earlier (Chottova Dvorakova et al., 2008).

### RNA Isolation

Whole tissue samples: total RNA was isolated from RA and LA and RV and LV of the heart (*n* = 7 per group) using the FastRNA Pro Green Kit (Qbiogene, Inc., CA) and FastPrep instrument (Qbiogene) according to the manufacturer’s instructions. LCM samples: total RNA was extracted with RNeasy Micro Kits (Qiagen, Hilden, Germany) following the protocol of manufacturer.

### Real-Time qPCR

Contaminating DNA was destroyed with 2 U of DNase (Invitrogen Corp., Carlsbad, CA, United States). First-strand cDNA was synthetized from 2 μg of total RNA in a 20 μl reaction mixture containing dithiothreitol, deoxynucleoside triphosphates, random primers, and Superscript RNase H-Reverse Transcriptase III (200 U/onset; Invitrogen Corp.) for 50 min at 42°C as described previously (Jarkovska et al., 2017). Real-time quantitative PCR was done in the I-Cycler (Bio-Rad, Munich, Germany) using primers and SYBR Green PCR kit (Bio-Rad). The primers used in the study are listed in **Table 1**. The PCR conditions were the following: initial denaturation in one cycle of 15 min at 95°C followed by 45 cycles of 30 s at 95°C, 23 s at 60°C or 64°C, and 30 s at 72°C. Although products emerged at a threshold of 20–37 cycles, a total of 50 cycles were run to enable quantitative analysis. All analyses were done in triplicate. Appropriate reaction conditions were determined by preparing and using plasmids containing corresponding sequence of bases as described previously (Slavikova et al., 2016). Freely available computational program BestKeeper was used for identifying the optimal normalization gene among a set of candidate reference genes, as it was explained in detail by the authors (Pfaffl et al., 2004). The most stable gene out of the tested reference genes – Hmbs, Ywhaz, and β-actin in our experimental conditions was β-actin (not shown), and therefore its expression level was used to normalize for differences in input cDNA.

**Table 1:** Characteristics of primer pairs used in RT-PCR.

Gene	*Range*	*Primer sequence*		Genebank Acc. No.
NK1R	869–1197 bp	For	GGTACTACGGCCTCT TCTATTGC	NM_012667
		Rev	CAGGAAGTAGATCAGT ACAGTCACG	
CRLR	789–929	For	CAACAGCACGCATGA GAAAGTG	NM_012717
		Rev	GTAATCCGTTGGCAAC TTAGGC	
RAMP1	157–293 bp	For	CATGGAGACCATAGG GAAGACT	AF181550
		Rev	AGAACTTGTCCACTTC CGGATTG	
Hmbs	908–1057 bp	For	GGCTGTAGCGTGCC AGTAGCAG	NM_013168.2
		Rev	TGGACCATCTTCTTGC TGAACA	
Ywhaz	650–782 bp	For	GATGAAGCCATTGCT GAACTTG	NM_013011.3
		Rev	CTCCCGCTTCTGCTTC GTCTCC	
β-actin	821–1029 bp	For	TTCCTTCCTGGGTATG GAATC	NM_031144
		Rev	GTTGGCATAGAGGTCT TTACGG	


*NK1R, neurokinin 1 receptor; CRLR, calcitonin-related like receptor; RAMP1, receptor activity modifying protein 1; Hmbs, hydroxymethylbilane synthase; Ywhaz, tyrosine 3-monooxygenase/tryptophan 5-monooxygenase activation protein, zeta polypeptide.*

The relative differences in gene abundance were calculated using quantitative cycle (Cq) values that were first normalized to those of the β-actin gene, and then relative to a control Cq value by the 2^-ΔΔ*C*_q_^ method (Livak and Schmittgen, 2001). Values obtained for group STZ26 were compared with those for Cont26, and STZ53 with Cont53, respectively. The PCR products were separated by electrophoresis on a 2.0% Tris-acetate-EDTA agarose gel. Control runs, where RT step or template was omitted, were negative.

### Western Blot

Preparation of extracts, sodium dodecyl sulfate (SDS) polyacrylamide gel electrophoresis, and western blotting were performed as described previously (Paddenberg et al., 2012). Per lane 25 μg protein extract was loaded. Primary antibodies used were rabbit anti-NK1R (Cat. No. LS-A1339; MBL International Corp., United States) diluted 1:1000 in Tris-buffered saline (TBS), 0.01% Tween 20, 5% milk powder and affinity-purified polyclonal anti-SDHA (1:5000 dilution), anti-SDHB (1:2000 dilution), or anti-SDHC antibody (1:15 000 dilution) (all descripted in Paddenberg et al., 2012). Secondary antibody used was peroxidase-conjugated goat anti-rabbit IgG antibody (Pierce, Rockford, IL, United States) diluted 1:10,000 in TBS, 0.01% Tween 20, 2.5% milk powder. For preabsorption control experiments, rabbit anti-NK1R antibody was mixed with a 6-fold, 3-fold, or 1.5-fold molar excess of the NK1R peptide (Cat. No LS-P1339; MBL International Corp., United States).

### Immunofluorescence

Shock frozen tissues from four hearts were cut into 10-μm-thick sections using a Leica 1900E cryostat (Leica, Bensheim, Germany). Sections were placed onto gelatinized slides, fixed for 20 min with acetone and allowed to air dry for 30 min at room temperature. Then they were covered for 1 h with blocking medium (PBS containing 0.1% bovine serum albumin, 10% normal porcine serum, and 0.5% Tween 20) and subsequently incubated with primary antibodies. The experiment was performed using rabbit polyclonal antisera against NK1R (1:50; Cat. No. LS-A1339; MBL International Corp., United States) alone or in combination with monoclonal antibody against RECA1 (1:20; Serotec, Düsseldorf, Germany) or SMA directly conjugated to FITC (1:100; Sigma, St. Louis, MO, United States), respectively. Antibodies were applied overnight at room temperature, followed by washing steps (2 × 10 min in PBS) and subsequent 1 h incubation with donkey-anti-rabbit IgG conjugated to TR (1:200; Chemicon, Temecula, CA, United States) or to FITC (1:250; Millipore, Temecula, CA, United States) and donkey-anti-mouse IgG conjugated to FITC (1:300; Dianova, Hamburg, Germany) or to Cy3 (1:1000; Millipore, Temecula, CA, United States) and PBS washes (2 × 10 min). Preabsorption of antisera with appropriate protein was done in order to prove specificity of binding. Finally, sections were covered in carbonate-buffered glycerol at pH 8.6. Evaluation was done by using an epifluorescence microscope (BX 60, Olympus, Prague, Czechia, or Axioplan 2 imaging, Zeiss, Jena, Germany) equipped with appropriate filter combinations.

### Laser Capture Microdissection

Shock frozen tissues from four hearts were cut into 8-μm-thick sections using a Leica 1900E cryostat (Leica, Bensheim, Germany). Sections were placed onto special slides (Zeiss, Jena, Germany) and stained with alum hematoxylin solution for 8 min and washed by water. Finally, the slides were dehydrated with 70% ethanol for 40 s and 100% ethanol for 40 s and subsequently air-dried. From the sections, intracardiac neurons, vascular smooth muscle cells, and cardiomyocytes were collected by use of microscope equipped with laser (Zeiss). About 60–80 cell profiles of one type were collected into one sample. Totally 15 samples containing neurons or smooth muscle or cardiomyocytes were collected. Subsequently RNA was isolated as described above.

### Statistical Analysis

All data are expressed as the mean ± standard error of the mean (SEM) and statistical analysis was performed using Mann–Whitney test. Values of *p* < 0.05 were considered statistically significant. The analysis was performed using the software package STATISTICA Cz, version 7 (StatSoft CR, Prague, Czechia) as mentioned previously (Jarkovska et al., 2017).

## Results

### RT-PCR

**FIGURE 1:**
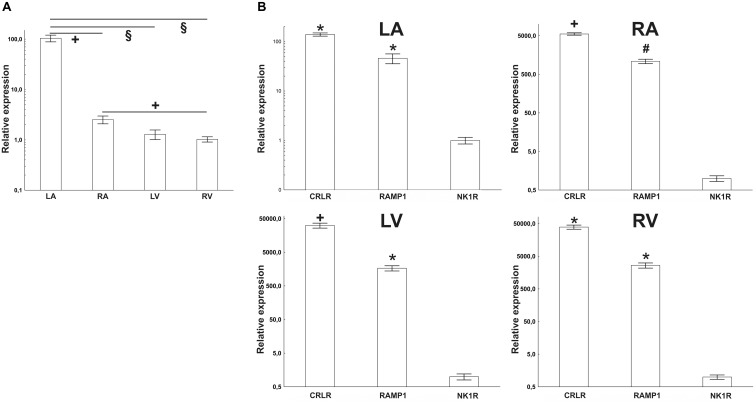
**(A)** RT-PCR quantification of NK1 receptor mRNAs in heart compartments (left atrium – LA, right atrium – RA, left ventricle – LV, and right ventricle – RV) of rats. Mean value from RV was used as a calibrator, when delta Cq value of this group was set as “1.” **(B)** Quantification of sensory receptors mRNAs by RT-PCR in heart compartments of rats; left atrium (LA), right atrium (RA), left ventricle (LV), and right ventricle (RV). Mean values of NK1 receptor (NK1R) expression were used as a calibrator. Expression of calcitonin receptor-like receptor (CRLR) and receptor activity modifying protein 1 (RAMP1) was compared to it. The difference is smallest in the LA and biggest in the RV. Data are presented as a relative expression ± SEM. ^§^ *p* < 0.0001, ^+^*p* < 0.05, ^#^*p* < 0.01, and ^∗^*p* < 0.005 (Mann–Whitney test) compared to corresponding heart compartment (in **A**) or compared to expression of NK1R (in **B**). *N* = 4–6 in each group.

In intact rat hearts, mRNA coding for the NK1R was observed in all heart chambers, RA, the LA, RV, and LV. Cq values were very high (they varied between 31 and 37), indicating that levels of the NK1R abundance were very low. As judged from ΔCq values, the NK1R mRNA abundance was the highest in LA. For the separate heart compartments, rank order of the NK1R relative abundance was LA > RA > RV = LV. LA contained about 100 times higher amount than RA and both ventricles (**Figure 1**). Very low levels of the NK1R mRNA in all heart compartments were also seen, when the abundances were compared with abundance of CRLR mRNA, another sensory receptor, and abundance of its receptor activity modifying protein (RAMP) 1 mRNA. The difference was the smallest in LA, where RAMP1 was expressed about 87 times more and CRLR about 140 times more than the abundance of NK1R (**Figure 1**).

### Western Blot

**FIGURE 2:**
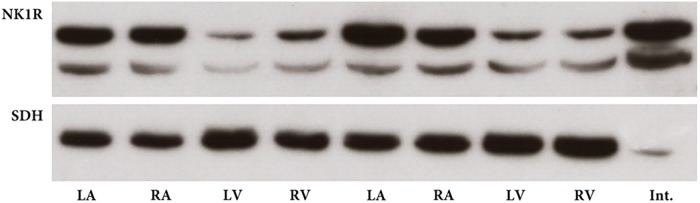
Western blot for NK1 receptor (NK1R) in heart compartments of two rats. NK1R immunoreactive band with expected molecular mass is distinctly present in all heart compartments, left atrium (LA), right atrium (RA), left ventricle (LV), and right ventricle (RV). Sample from intestine (Int.) serves as positive control. Succinate dehydrogenase A (SDH) immunoreactive bands with expected molecular mass demonstrate the presence of approximately similar amount of protein in each sample from heart compartments.

**FIGURE 3:**
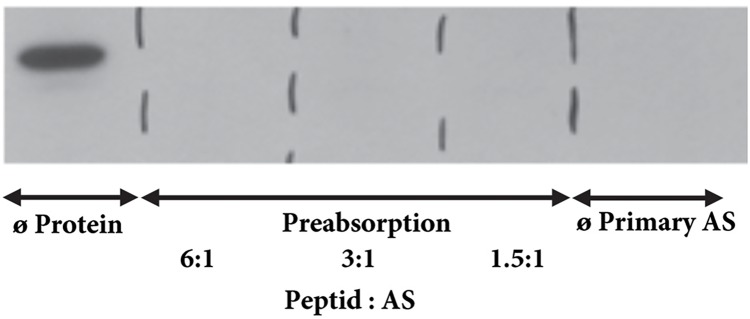
Western blot for NK1R – preabsorption. The NK1R immunoreactive band is detectable when primary antibody was not preabsorbed with corresponding peptide. No immunoreactive band is seen when primary antibody was pre-incubated with corresponding peptide with a 6-fold, 3-fold, or 1.5-fold molar excess of the NK1R peptide or when primary antibody is omitted.

Transcription of mRNA of the NK1R into protein was verified by western blotting which identified an immunoreactive protein in tissue extracts from each heart compartment. The specific band was clearly visible in all heart compartments being stronger in the atria than in ventricles (**Figure 2**). Overnight preabsorption of the primary antibody with corresponding synthetic peptide prevented recognition of the NK1R in all samples (**Figure 3**). So, it confirmed the monospecificity of the NK1R antiserum.

### Immunohistochemistry

**FIGURE 4:**
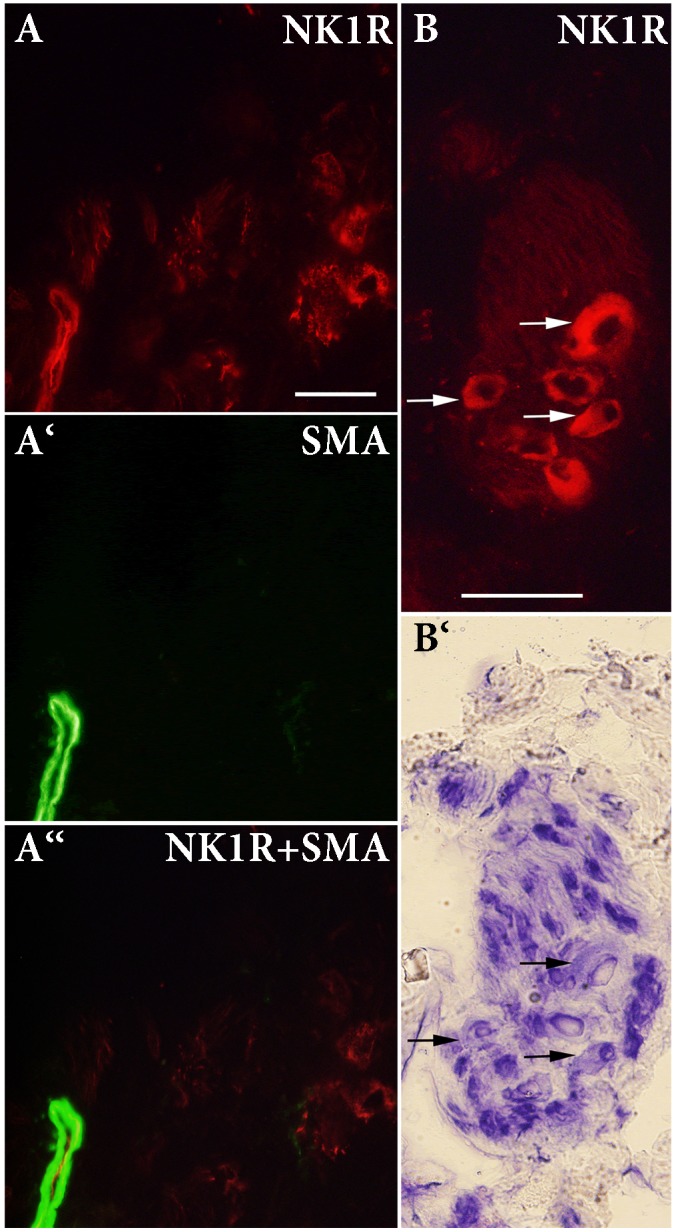
**(A,A‘,A“)** Double-labeling immunohistochemistry for NK1 receptor (NK1R) and smooth muscle actin (SMA) in the left atrium. A ganglion containing large-diameter neurons is shown. NK1R immunoreactivity (IR) is detected on the majority of intracardiac neurons and in smooth muscle cells. **(B,B‘)** IR for NK1R and histochemical staining of a neighboring frozen section to verify localization of NK1R-IR. NK1R-IR is seen in large-diameter neurons (arrows). Bar = 50 μm.

**FIGURE 5:**
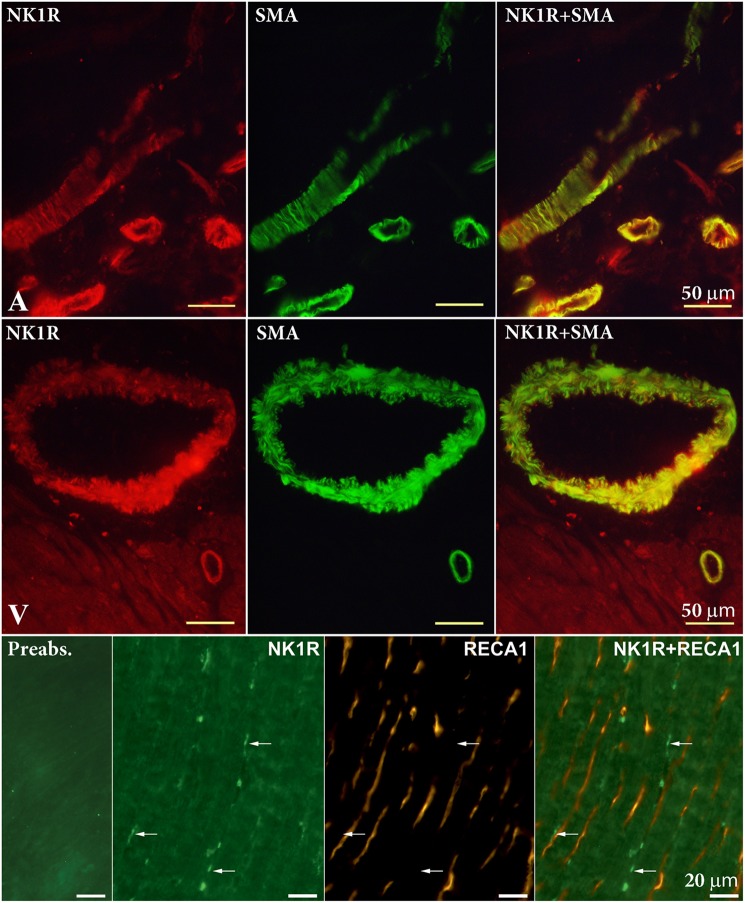
Double-labeling immunohistochemistry for NK1 receptor (NK1R) and smooth muscle actin (SMA) in the atrium (A) and ventricle (V), and rat endothelial cells antibody (RECA-1) in the V. Smooth muscle cells of arteries of different diameters exert NK1R-immunoreactivity. RECA-1 immunoreactive endothelial cells do not show any NK1R immunoreactivity. Anti-NK1R reacted with nerve fibers running between cardiomyocytes (arrows). Preabsorption with appropriate protein shows elimination of fluorescent signal.

To identify the localization of the NK1R in heart sections, we used specific antibodies against the NK1R, smooth muscle actin (SMA), and rat endothelial cells (RECA-1). NK1R-immunoreactivity (IR) was seen in large nerve cell bodies clustered in ganglia in sections of the LA. A cardiac ganglion was verified by the histochemical staining of a neighboring frozen section. In the same preparations, by the use of double labeling, both NK1R-IR and SMA-IR were observed in the wall of an artery and arterioles (**Figures 4**, **5**). Similar pattern of distribution of both NK1R-IR and SMA-IR in the wall of arteries with different diameter was also seen in the ventricles (**Figure 5**). Double labeling was also used to clarify position of NK1R-IR with respect to RECA-1-IR. The two positive stainings in the same preparation showed different localizations which are more clearly visible in the composite image (**Figure 5**). Presumably, anti-NK1R reacted with nerve fibers running between cardiomyocytes. Preabsorption of the primary antisera with corresponding synthetic peptide led to elimination of the fluorescent signal (**Figure 5**).

### Laser Capture Microdissection

**FIGURE 6:**
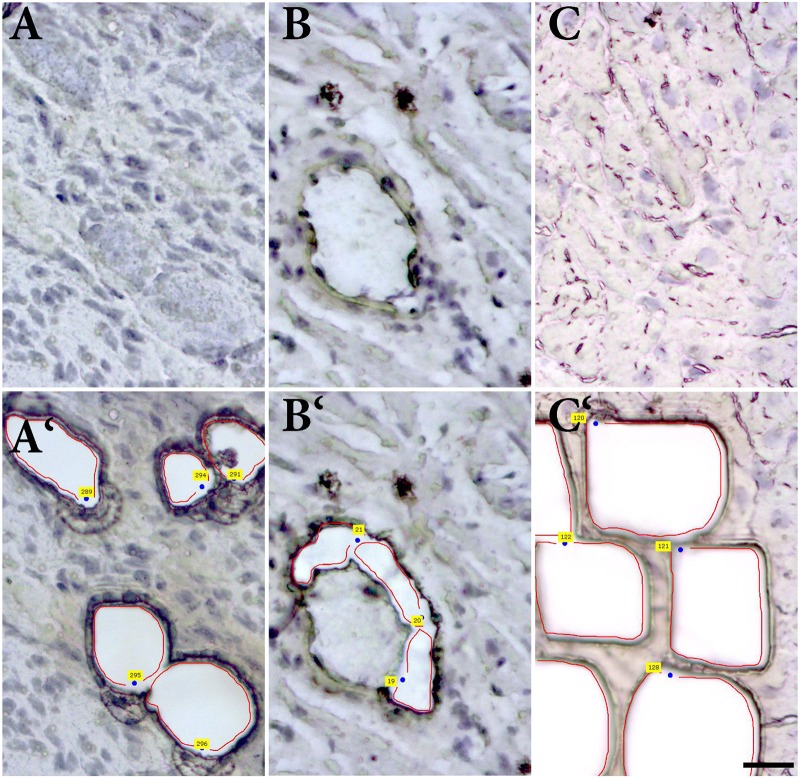
Laser-assisted microdissection. Tissue sections before and after the dissection of **(A,A‘)** ganglion cells, **(B,B‘)** smooth muscle cells of coronary artery, and **(C,C‘)** and cardiomyocytes.

**FIGURE 7:**
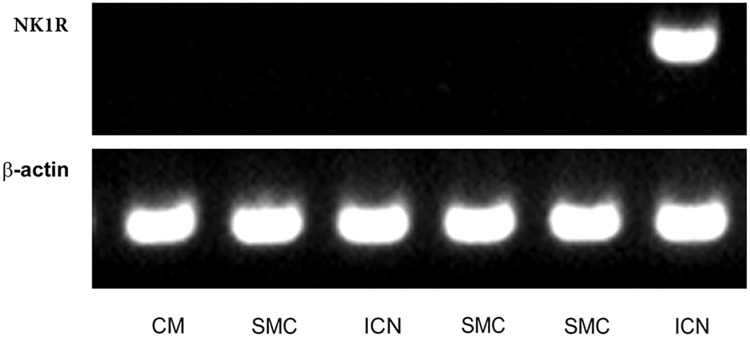
Laser-assisted microdissection. RT-PCR detection of mRNA for NK1 receptor (NK1R) and β-actin (control gene) in picked samples of cardiomyocytes (CM; *N* = 4), smooth muscle cells (SMC; *N* = 6), and intracardiac neurons (ICN; *N* = 5). Agarose gel electrophoresis of PCR products for β-actin (98 bp) and NK1R (329 bp).

Intracardiac neurons, smooth muscle cells form coronary artery wall, and cardiomyocytes were separately dissected (**Figure 6**) and analyzed. Cq values of the housekeeping gene (beta-actin) vary in range 25.5–32.8 in the samples, while no significant differences in Cq values were detected between particular cell types. NK1R mRNA was detectable in the two samples (out of five) of intracardiac neurons but not in any sample of other tested cell types (**Figure 7**).

### Diabetes Induction

**FIGURE 8:**
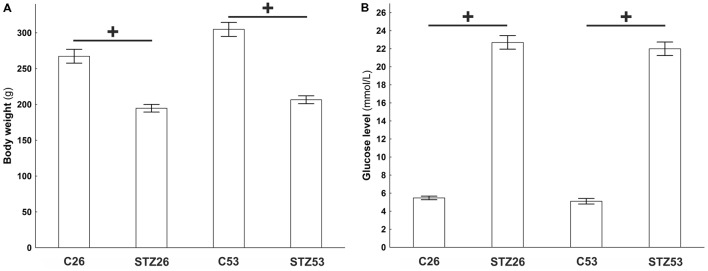
Body weight **(A)** and plasma glucose **(B)** of controls (C) and streptozotocin (STZ) treated animals measured in two time points, 26 and 53 weeks after the induction of diabetes. Data are presented as means ± SEMs. ^+^*p* < 0.0001. *N* = 10–16.

Within 1 week after injection of STZ, rats showed signs of diabetes that included hyperglycemia, weight loss, polyuria, and polydipsia. Just animals with blood glucose levels above 18 mmol/l were included in the study. Body weights of control and STZ-treated animals were significantly different (*p* < 0.001, *t*-test) at both time points analyzed (26 and 53 weeks after STZ injection; **Figure 8**).

**FIGURE 9:**
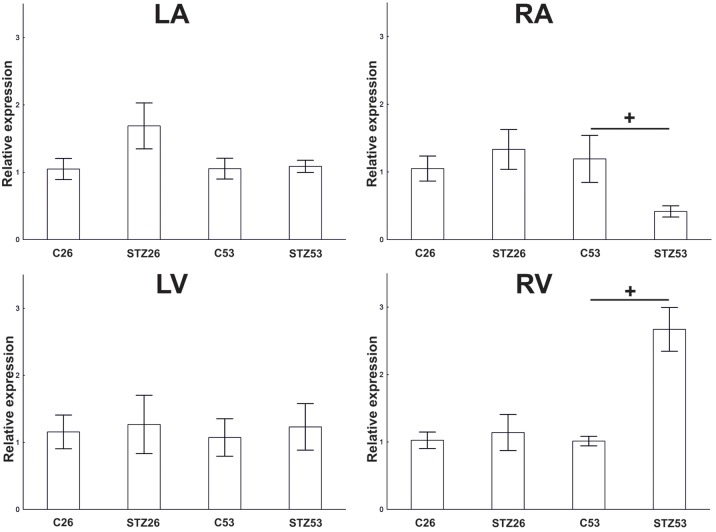
RT-PCR. Effect of STZ treatment on expression of NK1 receptor (NK1R) mRNA 26 and 53 weeks after the application of STZ in separated heart compartments, left atrium (LA), right atrium (RA), left ventricle (LV), and right ventricle (RV). Data are presented as relative expression values (compared to β-actin). Control values of the appropriate heart compartments were used as comparators and were settled as 1. The bars represent the means of the relative expression levels determined for each mRNA with standard errors of the mean. ^+^*p* < 0.05.

In the course of long-term diabetes, a significant downregulation of the NK1R mRNA was observed in the RA 53 weeks after the induction of diabetes. In contrast, at the same time point, NK1R mRNA abundance was markedly upregulated in the RV. No other significant differences were noted at other time points and locations within the heart, respectively (**Figure 9**).

## Discussion

Substance P is a sensory nerve neuropeptide. Previous reports showed that there are differences in the number and location of SP-containing nerves and the cellular distribution of SP binding sites in the heart among species. Especially, there is a lack of information related to the cellular distribution of the receptors (SP neurotransmission) in the rat heart. In both, the atrial and ventricular epicardium and myocardium, SP-containing fibers were identified in small amount in the rat compared to the guinea pig heart (Papka and Urban, 1987). In addition, a small population (5–10%) of coronary artery endothelial cells from the rat heart also contained SP (Milner et al., 1989). Within the nervous system in mammals, tachykinins are thought to act via three well-defined subclasses of NK receptors: NK1R, NK2R, and NK3R (Krause et al., 1993). Cardiovascular effects of SP are mediated by direct action on the cell-surface receptor NK1R, while NKA acts via the NK2R; however, there is some overlap between the two (Brain and Cox, 2006).

In our experiments, abundance of mRNA coding for the NK1R was detected in all heart compartments of control rats by RT-PCR. Transcription of this mRNA into NK1R protein was substantiated by the use of western blotting. We have identified an immunoreactive band of the expected molecular mass in all tissue extracts from each heart compartment. Thus, our results confirm that the specific binding sites for SP in the rat heart are localized in both atria and ventricles. The strongest bands have been detected in samples of the atrial preparations. Consistent with our findings, Dalsgaard et al. (1986) also detected many SP-containing fibers in the guinea pig atria but only few in the ventricles, which were mainly associated with blood vessels. Using radioimmunoassay analysis of heart samples from the guinea pig, they also revealed that amount of SP was about four times higher in the RA compared to the LV (Dalsgaard et al., 1986). In addition, we have revealed that relative expression of mRNA for NK1R was 100 times higher in the LA in comparison with the RA and the LV and RV. The highest gene abundance of the receptor in the LA may reflect association of these receptors with intracardiac ganglion cells since the majority of them is preferentially located within the LA and close to the interatrial septum (Pardini et al., 1987).

We have also examined the pattern of NK1R distribution by immunohistochemistry. In comparison with the single study published so far, in our experiments, positive immunostaining has a wider distribution. The NK1R-IR was detected in smooth muscle cells of coronary arteries and arterioles and further on certain cardiac ganglion neurons. Cardiac ganglia consist of heterogeneous population of afferent, efferent, interconnecting local circuit neurons and small intensely fluorescent cells. It is a biochemically and functionally very heterogeneous group of interconnected neurons containing both cholinergic and adrenergic neurons. These neurons contain in addition to classical mediator also one or more neuropeptides (Slavikova et al., 2003). All together are responsible for integration of sensory information from the heart with information coming from CNS (Kukanova and Mravec, 2006). Nerve fibers surrounding the neurons within the ganglion contain several neuropeptides including SP (Richardson et al., 2003). Additionally, SP-IR nerve fibers were demonstrated to be present closely to coronary vessels but absent between cardiac myocytes (Mistrova et al., 2016). No evidence was found to suggest the presence of NK1R on endothelial cells. In summary, this is the first study to demonstrate the expression of NK1R in the smooth muscle cells of vessels and cardiac ganglia cells in the rat heart. In the only study on the rat heart, Walsh et al. (1996) using radiography also observed SP binding sites over cells within clusters infrequently present in connective tissue between the heart and great vessels, which looks like those typical of connective tissue cells, e.g., macrophages and fibroblasts. Thus, localization of NK1R, which we demonstrated, corresponds to the direction of SP-containing axons within the heart as determined by immunohistochemistry, although the results of radiographic studies demonstrated the presence of binding sites for SP mainly in the connective tissue above the heart (Walsh et al., 1996) but also in intracardiac ganglia (Hoover and Hancock, 1988).

Our results fail to confirm the presence of the NK1R on cardiac muscle fibers. This finding is consistent with the results reported in the rat (Walsh et al., 1996) and guinea pig (Hoover and Hancock, 1988). However, isolated neonatal rat cardiomyocytes have been shown to possess NK1R and NK3R mRNAs, but not NK2R mRNA (Church et al., 1996). Recently, the presence of NK1R on isolated adult cardiomyocytes has been shown in rat (Jubair et al., 2015). Although our results and the results of other authors did not confirm the presence of NK1R in cardiomyocytes, data obtained by functional studies suggest a direct protective action of SP on cardiomyocytes by NK1R (Jubair et al., 2015). The explanation may lie in the low level of NKR1 abundance, which is sufficient to induce intracellular processes, but is below the detection limit of the methods used.

Relatively high abundance of NK1R mRNA within the left atria compared to right atria and both ventricles suggests neuronal cell bodies as a main source of it, while these cells are localized predominantly in the LA (Pardini et al., 1987). Using laser capture microdissection combined with quantitative RT-PCR, we have verified the abundance of NK1R mRNA in the intracardiac neurons. Additionally, we try to verify abundance of the mRNA in other cell types, arterial smooth muscle cells and cardiomyocytes; however, any of the samples did not contain detectable amount of tested mRNA. We would expect positive signal in samples with smooth muscle cells, while immunohistochemistry experiments demonstrate the presence of NK1R protein there. The absence of a PCR product in the samples may be caused by low abundance of the mRNA for this receptor in the cells.

In the course of long-term diabetes, 26 weeks after the STZ injection, there was no change in abundance of the gene for NK1R at in any compartment. However, at 53 weeks, the atria and ventricles responded differentially. A significant downregulation of NK1R mRNA was seen in the RA of STZ treated rats as compared to control animals, the atrial compartment with significantly lower relative abundance of the receptor in intact rats. In contrast, at the same time point, NK1R gene abundance was markedly upregulated in the RV. No significant changes were observed at other locations within the heart and time points, respectively. The reason that changes in the expression of the receptor appeared 1 year after the induction of diabetes can be that the damage to the individual tissues and organs in the course of diabetes occurs gradually. Some of the changes occurring in the innervation of the rat heart in relation to chronic diabetes have been already described. For example, neuropeptide Y receptor Y1 abundance has been found to increase in the rat heart only after 1 year of the duration of diabetes and not 26 or 39 weeks after the induction of diabetes (Chottova Dvorakova et al., 2008). SP signaling through NK1R is affected in several diseases whenever SP is involved. However, there are only a few studies suggesting changes in NK1R expression itself. A downregulation of mRNA for NK1R was observed in the dorsal root ganglia of diabetic rats (Boer et al., 2011). A decline of the abundance of SP and its receptor was also demonstrated in the atrial cardiomyocytes of diabetic patients compared to non-diabetic ones. A reduced abundance could be caused by impairment in the sensory nervous system since diabetic neuropathy damages the myocardial sensory nerve fibers (Ejaz et al., 2011a). The impaired expression of SP and its receptor may result in impaired ischemic revascularization (Kuo et al., 2007), impaired contractility (Khawaja and Rogers, 1996), and dysregulation in protein kinase C activation (Sieburth et al., 2007). Different effects of diabetes on the SP signaling in the atrial and ventricular myocardium have not been described yet. An upregulation of the NK1R abundance, as a phenomenon of sensitization response, however, might protect the heart from injury. Increased transcription of the NK1R gene in the diabetic heart may be induced by unidentified genes or factors involved in the development of diabetic cardiomyopathy. However, additional studies are needed to perform a more in-depth analysis of the SP signaling in different heart compartments in animal models of the long-term diabetes-associated cardiac dysfunction.

## Author Contributions

MCD, WK, and JS contributed to conception and design of the study. MCD and EM performed the experiments. MCD performed the statistical analysis. JS wrote the first draft of the manuscript. RP and MCD wrote sections of the manuscript. The figures were produced by MCD. All authors contributed to manuscript revision and read and approved the submitted version.

## Conflict of Interest Statement

The authors declare that the research was conducted in the absence of any commercial or financial relationships that could be construed as a potential conflict of interest.
